# The crystal structure of a new CdTe_2_O_5_ polymorph, isotypic with ∊-CaTe_2_O_5_

**DOI:** 10.1107/S2056989020006283

**Published:** 2020-05-15

**Authors:** Felix Eder, Matthias Weil

**Affiliations:** a Institute for Chemical Technologies and Analytics, Division of Structural Chemistry, TU Wien, Getreidemarkt 9/164-SC, A-1060 Vienna, Austria

**Keywords:** crystal structure, oxidotellurate(IV), cadmium tellurite, isotypism, structure comparison

## Abstract

Single crystals of cadmium penta­oxidoditellurate(IV), CdTe_2_O_5_, were obtained as by-products in a hydro­thermal reaction of Cd(NO_3_)_2_·4H_2_O, TeO_2_, H_6_TeO_6_ and NH_3_ (molar ratios 2:1:1:6) at 483 K for seven days. The crystals represent a different polymorph (henceforth referred to as the *β*-form) than the *α*-CdTe_2_O_5_ crystals grown from the melt, and are isotypic with hydro­thermally grown *∊*-CaTe_2_O_5_. The asymmetric unit of *β*-CdTe_2_O_5_ comprises one Cd, two Te and five O sites, all of which are located in general positions (Wyckoff position 4 *e*). The cadmium(II) atom is coordinated by seven oxygen atoms, forming ^2^_∞_[CdO_6/2_O_1/1_] (100) layers. Both tellurium sites are surrounded by four oxygen atoms with one of them being at a significantly longer distance than the other three. The resulting bis­phenoidal [TeO_4_] units also form layers propagating parallel to (100) by sharing edges with each other. The stereochemically active 5*s* ^2^ lone pair of the Te^IV^ atoms leads to the formation of large channels extending along [011] and smaller ones along [010]. A qu­anti­tative comparison between the crystal structures of *β*-CdTe_2_O_5_ and ∊-CaTe_2_O_5_ is made.

## Abstract 2

The crystal structure of a new polymorph of CdTe_2_O_5_, designated as the *β*-form, contains ^2^_∞_[Te_2_O_5_]^2–^ (100) layers with an undulating shape.

## Chemical context   

Cadmium penta­oxidoditellurate(IV), better known under its common name cadmium ditellurite, CdTe_2_O_5_, has been the subject of numerous investigations during the past decades with different emphases. In this regard, the CdO–TeO_2_ phase diagram was elucidated by Robertson *et al.* (1978 ▸), or the formation of glasses in the Cd–Te–O system by Karaduman *et al.* (2012 ▸). Other studies focused on electric and ferroelastic properties of CdTe_2_O_5_ (Redman *et al.*, 1970 ▸), with its ferro­elasticity remaining up to the melting point (Sadovskaya *et al.*, 1983 ▸; Gorbenko *et al.*, 1990 ▸). Single crystals of CdTe_2_O_5_ are usually grown from the melt utilizing the Czochralski or Bridgeman techniques as crystal growth methods (Nawash, 2015 ▸). Even though single crystals of CdTe_2_O_5_ have been grown for decades this way, a satisfactory structure model for this phase had never been published so far, and only lattice parameters of a sub-cell were given (Redman *et al.*, 1970 ▸). Other phases in the Cd–Te–O system that are compiled in the Inorganic Crystal Structure Database (ICSD; Zagorac *et al.*, 2019 ▸) include two polymorphs of CdTe^IV^O_3_ (Krämer & Brandt, 1985 ▸; Poupon *et al.*, 2017 ▸), two polymorphs of Cd_3_Te^VI^O_6_ (Burckhardt *et al.*, 1982 ▸; Weil & Veyer, 2018 ▸) and the mixed Te^IV/VI^-compounds Cd_2_Te_2_O_7_ and Cd_2_Te_3_O_9_ (Weil, 2004 ▸).

The lack of a reasonable structure model for CdTe_2_O_5_ might be caused by the micaceous appearance of the grown crystals (Redman *et al.*, 1970 ▸), as well as by its ferroelastic properties, which often are correlated with the formation of twins or multiple domains. The new CdTe_2_O_5_ phase discovered during the present study originally intended to synthesize new mixed-valent cadmium oxidotellurates(IV,VI), however, belongs to a different polymorph, hereafter referred to as the *β*-form of CdTe_2_O_5_.

In this communication we report on the synthesis and crystal structure analysis of *β*-CdTe_2_O_5_ and compare it quan­ti­tatively with the isotypic structure of *∊*-CaTe_2_O_5_ (Weil & Stöger, 2008 ▸; Barrier *et al.*, 2009 ▸).

## Structural commentary   

**Figure 1:**
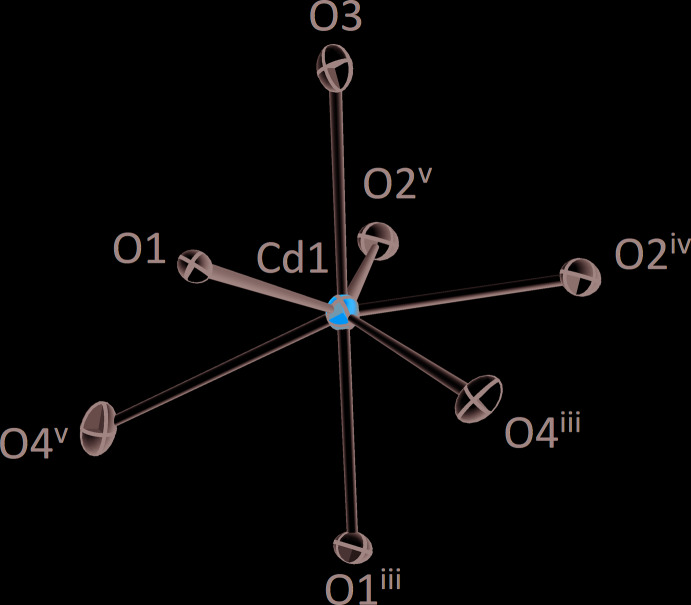
The [CdO_7_] polyhedron in the crystal structure of *β*-CdTe_2_O_5_. Displacement ellipsoids are drawn at the 90% probability level. Symmetry codes refer to Table 1 ▸.

All atoms in the asymmetric unit, *viz*. one Cd site, two Te sites and five O sites, are located on general Wyckoff positions 4 *e* (site symmetry 1). The cadmium atom is coordinated by seven oxygen atoms with distances in a range of 2.235 (3)–2.688 (3) Å. The average Cd—O bond length is 2.389 Å, which is in accordance with the sum of ionic radii for Cd^II^ (CN 7; 1.17 Å) and O (CN 3; 1.22 Å) compiled by Shannon (1976 ▸). The bond-valence sum (BVS; Brown, 2002 ▸) of Cd is 2.07 valence units (v.u.) using the values of Brese & O’Keeffe (1991 ▸) for calculation. The [CdO_7_] polyhedron is best described as a distorted penta­gonal bipyramid (Fig. 1 ▸). The [CdO_7_] polyhedra are connected to each other by sharing three edges with other [CdO_7_] units, thereby forming ^2^_∞_[CdO_6/2_O_1/1_] layers oriented parallel to (100) with the Cd^II^ atoms located at *x* ≃ 0;1.

**Figure 2:**
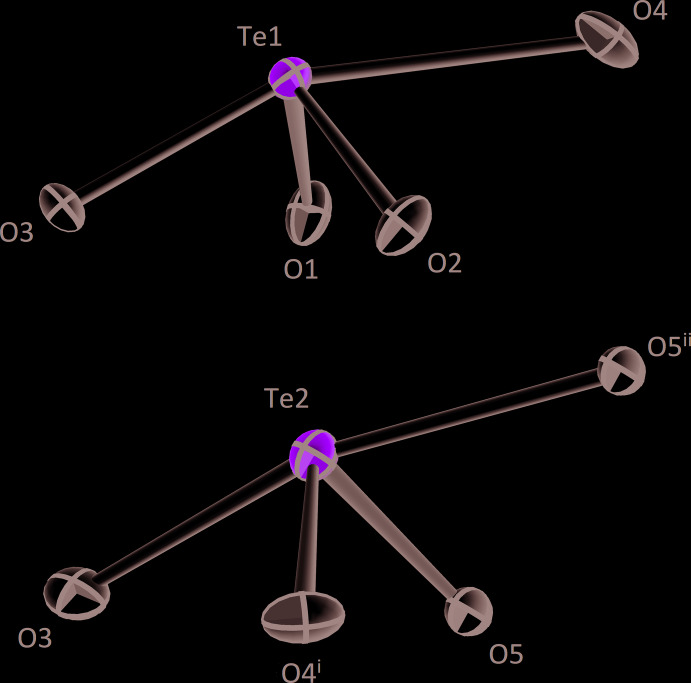
The [TeO_4_] polyhedra in the crystal structure of *β*-CdTe_2_O_5_. Displacement ellipsoids are drawn at the 90% probability level. Symmetry codes refer to Table 1 ▸.

The two tellurium(IV) atoms are both coordinated by four oxygen atoms with three of them being closer than 2 Å (Table 1 ▸) and the fourth one at a distance of 2.285 (3) Å (Te1) and 2.204 (3) Å (Te2), respectively. The oxygen atoms are located to one side of the Te^IV^ atoms due to the large amount of space the 5*s* ^2^ electron lone pair requires. The corresponding coordination polyhedra can be derived from a trigonal bipyramid, [ΨTeO_4_], with the lone pair occupying one of the equatorial positions in each case. The shapes of the polyhedra without the contribution of the lone pair correspond to [TeO_4_] bis­phenoids (Fig. 2 ▸). The bond-valence sums for the tellurium(IV) atoms were calculated to be 4.15 and 4.10 v.u. for Te1 and Te2, respectively, using the values of Brese & O’Keeffe (1991 ▸). When applying the revised parameters of Mills & Christy (2013 ▸), BVS of 3.94 and 3.92 v.u. were obtained.

**Figure 3:**
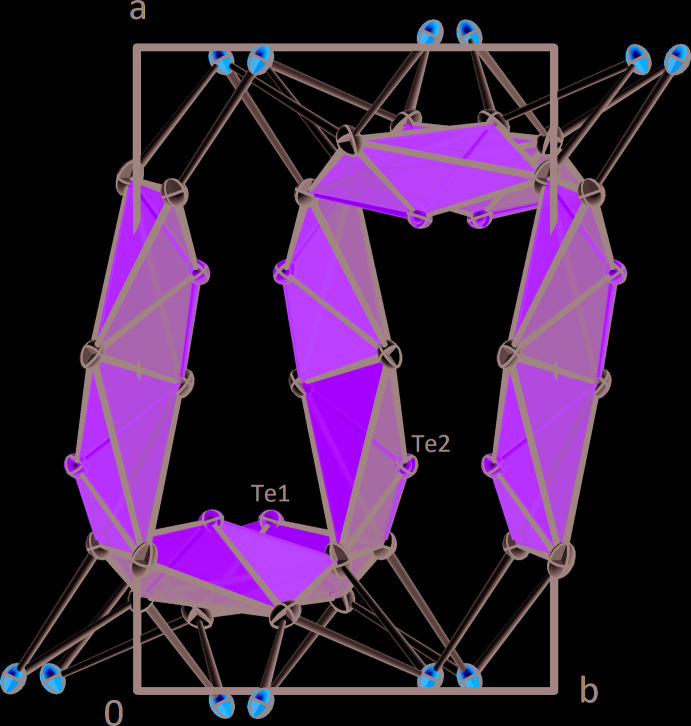
The crystal structure of *β*-CdTe_2_O_5_ in a projection along [001]. Displacement ellipsoids are drawn at the 90% probability level.

**Figure 4:**
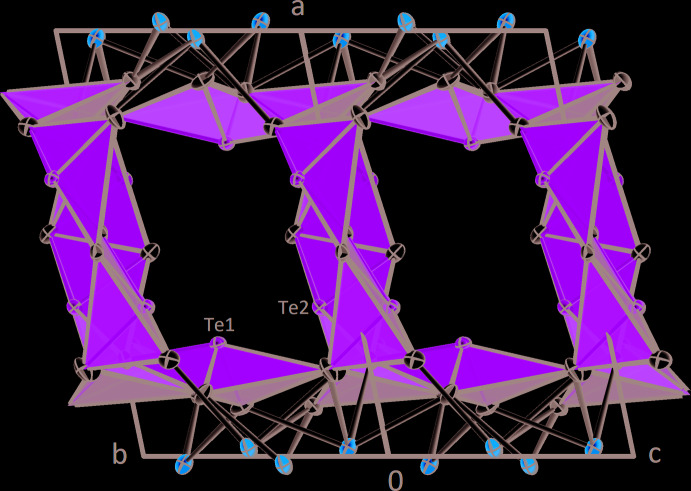
Large channels in the *β*-CdTe_2_O_5_ structure running parallel to [011]. Displacement ellipsoids are drawn at the 90% probability level.

**Figure 5:**
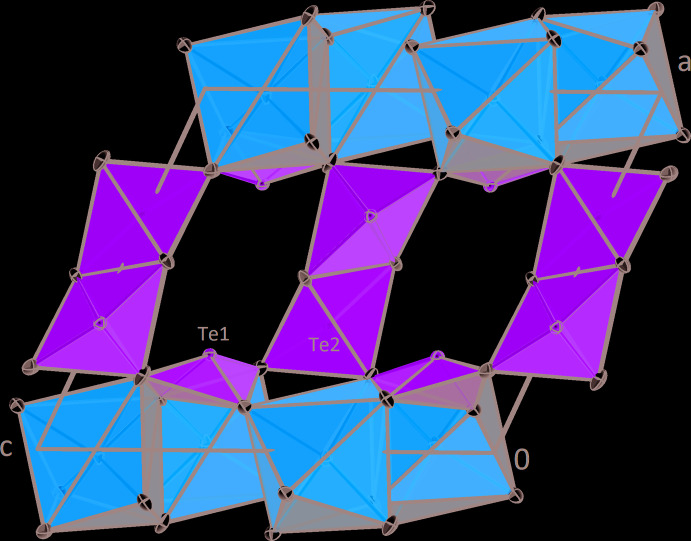
Smaller channels in the *β*-CdTe_2_O_5_ structure running parallel to [010]. Displacement ellipsoids are drawn at the 90% probability level.

The [TeO_4_] polyhedra are connected to each other to form layers oriented parallel to (100). These layers have a distinct undulating shape (Fig. 3 ▸) and are built up by chains of [Te1O_4_] and [Te2O_4_] units arranged alternately by sharing corners with two neighbours. These chains are cross-linked by two [Te2O_4_]-polyhedra by sharing an edge consisting of two O5 atoms. The [Te1O_4_] units are located very close to the Cd–O layer and share edges with three [CdO_7_] polyhedra and one corner with a fourth one. The [Te2O_4_] units are positioned in the centre of the layer and only share two corners with three [CdO_7_] polyhedra. The rather loose arrangement of [TeO_4_] units in the layer can be explained by the stereochemically active 5*s* ^2^ electron lone pair situated at each of the two Te^IV^ atoms. The space requirements of the non-bonding electron pairs lead to the undulating shape of the layer, which results in the presence of large channels in the structure, which are oriented parallel to [011] (Fig. 4 ▸). Smaller channels are also realized and propagate parallel to [010] (Fig. 5 ▸).

The arrangement of such an undulating ^2^_∞_[Te_2_O_5_]^2–^ layer was reported for the first time for the *∊*-polymorph of CaTe_2_O_5_ (Weil & Stöger, 2008 ▸), which is isotypic with *β*-CdTe_2_O_5_. CaTe_2_O_5_ is likewise reported to crystallize in a mica-like form from the melt (Redman *et al.*, 1970 ▸). Although several high-temperature polymorphs have also been reported for this phase (Tripathi *et al.*, 2001 ▸), ∊-CaTe_2_O_5_ is the only polymorph for which a crystal-structure determination has been performed (Weil & Stöger, 2008 ▸; Barrier *et al.*, 2009 ▸). The close similarity between the two structures can be explained by the very similar ionic radii (Shannon, 1976 ▸) of Ca (CN 7: 1.20 Å) and Cd (CN 7: 1.17 Å). The corresponding bond lengths in the isotypic structures (Table 1 ▸) differ only slightly with one exception: the Te1—O4 bond, which is 0.165 Å longer in the Ca structure, shows by far the biggest difference. A qu­anti­tative comparison between *β*-CdTe_2_O_5_ and *∊*-CaTe_2_O_5_ was carried out using the *compstru* software (de la Flor *et al.*, 2016 ▸), available at the Bilbao Crystallographic Server (Aroyo *et al.*, 2006 ▸). The absolute distances between paired atoms are 0.0303 Å for Cd/Ca1, 0.0628 Å for Te1, 0.0178 Å for Te2, 0.1426 Å for O1, 0.0791 Å for O2, 0.0384 Å for O3, 0.0788 Å for O4 and 0.0635 Å for O5. The degree of lattice distortion is 0.0118, the arithmetic mean of the distance between paired atoms is 0.0642 Å, and the measure of similarity is 0.077.

## Synthesis and crystallization   

Crystals of CdTe_2_O_5_ were obtained under hydro­thermal conditions. The reactants, 0.1890 g (0.613 mmol) Cd(NO_3_)_3_·2H_2_O, 0.0484 g (0.303 mmol) TeO_2_, 0.0710 g (0.309 mmol) H_6_TeO_6_ and 0.12 g (1.8 mmol) 25%_wt_ NH_3(aq)_ were weighed into a small teflon vessel with a volume of *ca* 3 ml. Deionized water was added until the vessel was filled to about two thirds of its volume. Then the vessel was heated to 483 K in a steel autoclave for 7 d under autogenous pressure. Afterwards, the autoclave was cooled to room temperature within about 4 h. The reaction product was a light-yellow, almost white solid. In the X-ray powder pattern of the bulk, *α*-Cd_3_TeO_6_ (Burckhardt *et al.*, 1982 ▸) and *β*-CdTe_2_O_5_ were found. Under a polarising microscope a few small shiny colourless blocks of *β-*CdTe_2_O_5_ were isolated for single-crystal measurements.

## Refinement   

Crystal data, data collection and structure refinement details are summarized in Table 2 ▸. Atom labels and starting coord­inates for refinement were adopted from the isotypic *∊*-CaTe_2_O_5_ structure (Weil & Stöger, 2008 ▸).

## Supplementary Material

Crystal structure: contains datablock(s) I. DOI: 10.1107/S2056989020006283/hb7913sup1.cif

Structure factors: contains datablock(s) I. DOI: 10.1107/S2056989020006283/hb7913Isup2.hkl

CCDC reference: 2002758

Additional supporting information:  crystallographic information; 3D view; checkCIF report

## Figures and Tables

**Table 1:** Comparison of Te—O and *M*—O (*M* = Cd, Ca) bond lengths (Å) in the isotypic *β*-CdTe_2_O_5_ and *∊*-CaTe_2_O_5_ structures

	*β*-CdTe_2_O_5_	*∊*-CaTe_2_O_5_^*a*^
Te1—O2	1.843 (3)	1.832 (4)
Te1—O1	1.875 (3)	1.852 (4)
Te1—O3	1.991 (3)	1.980 (5)
Te1—O4	2.285 (3)	2.450 (5)
Te2—O4^i^	1.864 (3)	1.854 (4)
Te2—O5	1.897 (3)	1.898 (4)
Te2—O3	1.990 (3)	2.009 (5)
Te2—O5^ii^	2.204 (5)	2.178 (5)
*M*1—O1^iii^	2.235 (3)	2.305 (4)
*M*1—O2^iv^	2.238 (3)	2.326 (4)
*M*1—O1	2.256 (3)	2.360 (5)
*M*1—O2^v^	2.296 (3)	2.358 (5)
*M*1—O3	2.424 (3)	2.476 (5)
*M*1—O4^v^	2.589 (3)	2.554 (5)
*M*1—O4^iii^	2.688 (3)	2.682 (5)

(*a*) Lattice parameters: *a* = 9.382 (2), *b* = 5.7095 (14), *c* = 11.132 (3) Å, *β* = 115.109 (4)°, *V* = 539.95 Å^3^ (Weil & Stöger, 2008 ▸). [Symmetry codes: (i) *x*, −*y* + 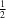, *z* − 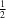; (ii) −*x* + 1, −*y* + 1, −*z* + 1; (iii) −*x*, *y* + 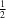, −*z* + 1; (iv) −*x*, −*y* + 1, −*z* + 1; (v) *x*, *y* + 1, *z*.]

**Table 2:** Experimental details

Crystal data	
Chemical formula	CdTe_2_O_5_
*M*_r_	447.60
Crystal system, space group	Monoclinic, *P*2_1_/*c*
Temperature (K)	100
*a*, *b*, *c* (Å)	9.4535 (5), 5.5806 (3), 10.8607 (5)
β (°)	114.430 (1)
*V* (Å^3^)	521.67 (5)
*Z*	4
Radiation type	Mo *K*α
μ (mm^−1^)	15.08
Crystal size (mm)	0.10 × 0.06 × 0.05

Data collection	
Diffractometer	Bruker APEXII CCD
Absorption correction	Multi-scan (*SADABS*; Krause *et al.*, 2015 ▸)
*T*_min_, *T*_max_	0.600, 0.746
No. of measured, independent and observed [*I* > 2σ(*I*)] reflections	9236, 1827, 1462
*R*_int_	0.045
(sin θ/λ)_max_ (Å^−1^)	0.747

Refinement	
*R*[*F* ^2^ > 2σ(*F* ^2^)], *wR*(*F* ^2^), *S*	0.024, 0.045, 1.00
No. of reflections	1827
No. of parameters	73
Δρ_max_, Δρ_min_ (e Å^−3^)	1.20, −1.04

Computer programs: *APEX3* (Bruker, 2016 ▸), *SAINT* (Bruker, 2016 ▸), *SHELXT* (Sheldrick, 2015*a* ▸), *SHELXL* (Sheldrick, 2015*b* ▸), *ATOMS* (Dowty, 2006 ▸), *publCIF* (Westrip, 2010 ▸).

## supplementary crystallographic information

### Crystal data

**Table:**

CdTe_2_O_5_	*F*(000) = 768
*M**_r_* = 447.60	*D*_x_ = 5.699 Mg m^−^^3^
Monoclinic, *P*2_1_/*c*	Mo *K*α radiation, λ = 0.71073 Å
*a* = 9.4535 (5) Å	Cell parameters from 2645 reflections
*b* = 5.5806 (3) Å	θ = 2.4–32.1°
*c* = 10.8607 (5) Å	µ = 15.08 mm^−^^1^
β = 114.430 (1)°	*T* = 100 K
*V* = 521.67 (5) Å^3^	Block, colourless
*Z* = 4	0.10 × 0.06 × 0.05 mm

### Data collection

**Table:**

Bruker APEXII CCD diffractometer	1462 reflections with *I* > 2σ(*I*)
ω– and φ–scan	*R*_int_ = 0.045
Absorption correction: multi-scan (*SADABS*; Krause *et al.*, 2015)	θ_max_ = 32.1°, θ_min_ = 3.8°
*T*_min_ = 0.600, *T*_max_ = 0.746	*h* = −14→14
9236 measured reflections	*k* = −8→8
1827 independent reflections	*l* = −15→16

### Refinement

**Table:**

Refinement on *F*^2^	0 restraints
Least-squares matrix: full	Primary atom site location: isomorphous structure methods
*R*[*F*^2^ > 2σ(*F*^2^)] = 0.024	*w* = 1/[σ^2^(*F*_o_^2^) + (0.0171*P*)^2^] where *P* = (*F*_o_^2^ + 2*F*_c_^2^)/3
*wR*(*F*^2^) = 0.045	(Δ/σ)_max_ = 0.001
*S* = 1.00	Δρ_max_ = 1.20 e Å^−^^3^
1827 reflections	Δρ_min_ = −1.03 e Å^−^^3^
73 parameters	

### Special details

**Table:**

Geometry. All esds (except the esd in the dihedral angle between two l.s. planes) are estimated using the full covariance matrix. The cell esds are taken into account individually in the estimation of esds in distances, angles and torsion angles; correlations between esds in cell parameters are only used when they are defined by crystal symmetry. An approximate (isotropic) treatment of cell esds is used for estimating esds involving l.s. planes.

### Fractional atomic coordinates and isotropic or equivalent isotropic displacement parameters (Å^2^)

**Table:**

	*x*	*y*	*z*	*U*_iso_*/*U*_eq_	
Te1	0.25911 (3)	0.32034 (5)	0.72297 (3)	0.01195 (7)	
Te2	0.34935 (3)	0.64780 (5)	0.49078 (3)	0.01181 (7)	
Cd1	0.01900 (4)	0.79762 (6)	0.63620 (3)	0.01416 (8)	
O1	0.1293 (4)	0.4948 (5)	0.7821 (3)	0.0141 (6)	
O2	0.1216 (4)	0.1359 (5)	0.5843 (3)	0.0188 (7)	
O3	0.2239 (4)	0.5960 (5)	0.5973 (3)	0.0163 (7)	
O4	0.2081 (4)	0.0287 (6)	0.8466 (3)	0.0162 (7)	
O5	0.4791 (4)	0.3979 (6)	0.5962 (3)	0.0176 (7)	

### Atomic displacement parameters (Å^2^)

**Table:**

	*U*^11^	*U*^22^	*U*^33^	*U*^12^	*U*^13^	*U*^23^
Te1	0.00997 (13)	0.01304 (14)	0.01191 (13)	0.00123 (10)	0.00360 (10)	−0.00069 (10)
Te2	0.01214 (13)	0.01187 (13)	0.01070 (13)	−0.00178 (10)	0.00399 (10)	−0.00077 (10)
Cd1	0.01824 (16)	0.01311 (16)	0.01254 (15)	0.00370 (12)	0.00778 (13)	0.00255 (12)
O1	0.0171 (16)	0.0124 (15)	0.0175 (15)	0.0035 (12)	0.0116 (13)	0.0054 (12)
O2	0.0256 (18)	0.0146 (16)	0.0109 (15)	−0.0055 (14)	0.0023 (13)	−0.0008 (13)
O3	0.0199 (17)	0.0142 (15)	0.0209 (16)	0.0035 (13)	0.0144 (14)	0.0032 (13)
O4	0.0162 (16)	0.0142 (15)	0.0159 (15)	0.0016 (13)	0.0044 (13)	0.0068 (13)
O5	0.0162 (16)	0.0198 (16)	0.0188 (16)	0.0060 (13)	0.0094 (13)	0.0072 (13)

### Geometric parameters (Å, º)

**Table:**

Te1—O2	1.843 (3)	Te2—Te2^ii^	3.2253 (6)
Te1—O1	1.875 (3)	Cd1—O1^iii^	2.235 (3)
Te1—O3	1.991 (3)	Cd1—O2^iv^	2.238 (3)
Te1—O4	2.285 (3)	Cd1—O2^v^	2.296 (3)
Te2—O4^i^	1.864 (3)	Cd1—O1	2.255 (3)
Te2—O5	1.897 (3)	Cd1—O3	2.424 (3)
Te2—O3	1.990 (3)	Cd1—O4^v^	2.589 (3)
Te2—O5^ii^	2.204 (3)	Cd1—O4^iii^	2.688 (3)
			
O2—Te1—O1	103.25 (15)	O1^iii^—Cd1—O3	167.39 (11)
O2—Te1—O3	90.59 (13)	O2^iv^—Cd1—O3	93.09 (12)
O1—Te1—O3	83.46 (12)	O2^v^—Cd1—O3	83.66 (11)
O2—Te1—O4	80.40 (12)	O1—Cd1—O3	66.67 (10)
O1—Te1—O4	80.89 (12)	O1^iii^—Cd1—O4^v^	73.85 (11)
O3—Te1—O4	159.60 (12)	O2^iv^—Cd1—O4^v^	138.57 (10)
O4^i^—Te2—O5	100.25 (14)	O2^v^—Cd1—O4^v^	66.40 (10)
O4^i^—Te2—O3	91.11 (13)	O1—Cd1—O4^v^	78.65 (10)
O5—Te2—O3	86.24 (13)	O3—Cd1—O4^v^	94.31 (10)
O4^i^—Te2—O5^ii^	88.71 (13)	Te1—O1—Cd1^vi^	119.15 (14)
O5—Te2—O5^ii^	76.52 (13)	Te1—O1—Cd1	109.06 (13)
O3—Te2—O5^ii^	162.43 (12)	Cd1^vi^—O1—Cd1	117.64 (13)
O4^i^—Te2—Te2^ii^	95.12 (10)	Te1—O2—Cd1^iv^	133.44 (16)
O5—Te2—Te2^ii^	41.63 (9)	Te1—O2—Cd1^vii^	119.05 (14)
O3—Te2—Te2^ii^	127.79 (9)	Cd1^iv^—O2—Cd1^vii^	105.95 (12)
O5^ii^—Te2—Te2^ii^	34.89 (8)	Te1—O3—Te2	122.67 (15)
O1^iii^—Cd1—O2^iv^	98.69 (12)	Te1—O3—Cd1	99.09 (12)
O1^iii^—Cd1—O2^v^	95.20 (12)	Te2—O3—Cd1	138.18 (14)
O2^iv^—Cd1—O2^v^	74.05 (13)	Te2^viii^—O4—Te1	128.18 (16)
O1^iii^—Cd1—O1	105.89 (7)	Te2^viii^—O4—Cd1^vii^	118.13 (14)
O2^iv^—Cd1—O1	140.67 (11)	Te1—O4—Cd1^vii^	94.15 (10)
O2^v^—Cd1—O1	131.97 (12)	Te2—O5—Te2^ii^	103.48 (13)

Symmetry codes: (i) *x*, −*y*+1/2, *z*−1/2; (ii) −*x*+1, −*y*+1, −*z*+1; (iii) −*x*, *y*+1/2, −*z*+3/2; (iv) −*x*, −*y*+1, −*z*+1; (v) *x*, *y*+1, *z*; (vi) −*x*, *y*−1/2, −*z*+3/2; (vii) *x*, *y*−1, *z*; (viii) *x*, −*y*+1/2, *z*+1/2.
